# Exploring lncRNA-Mediated Regulatory Networks in Endometrial Cancer Cells and the Tumor Microenvironment: Advances and Challenges

**DOI:** 10.3390/cancers11020234

**Published:** 2019-02-16

**Authors:** Peixin Dong, Ying Xiong, Junming Yue, Sharon J. B. Hanley, Noriko Kobayashi, Yukiharu Todo, Hidemichi Watari

**Affiliations:** 1 Department of Obstetrics and Gynecology, Hokkaido University School of Medicine, Hokkaido University, Sapporo 060-8638, Japan; sjbh1810@mta.biglobe.ne.jp (S.J.B.H.); norikingyo@med.hokudai.ac.jp (N.K.); 2 Department of Gynecology, State Key Laboratory of Oncology in South China, Sun Yat-sen University Cancer Center, Guangzhou 510060, China; tdken999@163.com; 3 Department of Pathology and Laboratory Medicine, University of Tennessee Health Science Center, Memphis, TN 38163, USA; jyue@uthsc.edu; 4 Center for Cancer Research, University of Tennessee Health Science Center, Memphis, TN 38163, USA; 5 Division of Gynecologic Oncology, National Hospital Organization, Hokkaido Cancer Center, Sapporo 003-0804, Japan; yukiharu@sap-cc.go.jp

**Keywords:** long non-coding RNA, microRNA, endometrial cancer, prognostic biomarker, therapeutic target, epigenetics, regulatory mechanism, tumor microenvironment

## Abstract

Recent studies have revealed both the promise and challenges of targeting long non-coding RNAs (lncRNAs) to diagnose and treat endometrial cancer (EC). LncRNAs are upregulated or downregulated in ECs compared to normal tissues and their dysregulation has been linked to tumor grade, FIGO stage, the depth of myometrial invasion, lymph node metastasis and patient survival. Tumor suppressive lncRNAs (GAS5, MEG3, FER1L4 and LINC00672) and oncogenic lncRNAs (CCAT2, BANCR, NEAT1, MALAT1, H19 and Linc-RoR) have been identified as upstream modulators or downstream effectors of major signaling pathways influencing EC metastasis, including the PTEN/PI3K/AKT/mTOR, RAS/RAF/MEK/ERK, WNT/β-catenin and p53 signaling pathways. TUG1 and TDRG1 stimulate the VEGF-A pathway. PCGEM1 is implicated in activating the JAK/STAT3 pathway. Here, we present an overview of the expression pattern, prognostic value, biological function of lncRNAs in EC cells and their roles within the tumor microenvironment, focusing on the influence of lncRNAs on established EC-relevant pathways. We also describe the emerging classification of EC subtypes based on their lncRNA signature and discuss the clinical implications of lncRNAs as valuable biomarkers for EC diagnosis and potential targets for EC treatment.

## 1. Introduction

Endometrial cancer (EC) arises from the lining of the uterus and is the most common gynecologic cancer in the developed world [1]. Although the majority of patients with endometrial cancer (EC) have good outcomes, advanced or metastatic disease carries a grave prognosis [2]. Thus, this highlights the need to identify biomarkers that would distinguish aggressive from indolent disease and for developing novel therapies to treat EC. 

EC has been broadly classified into two groups with distinct clinical and molecular alterations [2,3]. Type I EC is usually low-grade, low-stage endometrioid adenocarcinomas with favorable prognosis [2,3]. The most important altered pathway in type I EC is the PTEN/PI3K/AKT/mTOR pathway [2,3]. The aberrant activation of the RAS/RAF/MEK/ERK and WNT/β-catenin signaling pathway is also common in type I EC, [2,4]. Type II EC is often high-grade endometrioid adenocarcinomas and serous carcinomas with worse prognosis, which harbor TP53 mutations and HER2 overexpression [2,3]. The upregulation of EGFR and VEGF-A is found in both type I and II EC [5,6]. However, this dualistic model has limitations because the significant heterogeneity within and the overlapping features between Type I and II EC have been recognized [7]. 

The Cancer Genome Atlas (TCGA) has reclassified EC into four genomic subgroups: the ultra-mutated group harboring mutations in the exonuclease domain of POLE gene (POLE ultra-mutated), microsatellite instability, copy number-low as well as copy number-high and serous-like subgroups [8]. However, the copy number-low group (around 40% of all EC patients with no specific molecular profile) still represents a poorly characterized heterogeneous set of EC. 

The human genome is pervasively transcribed and most of the human transcriptome is composed of non-coding RNAs (ncRNAs) [9]. Based on transcript size, the long non-coding RNAs (lncRNAs) are defined as transcripts of greater than 200 nucleotides. LncRNAs have been classified as exonic lncRNAs, intronic lncRNAs, intergenic lncRNAs overlapping lncRNAs and anti-sense lncRNAs [10]. 

A subset of lncRNAs is present in the nucleus, but the majority of lncRNA is enriched in the cytoplasm or is located in both the nucleus and cytoplasm [11]. Although only a few lncRNAs have been fully investigated, it is clear that lncRNAs are expressed in a tissue-specific manner and can act as upstream modulators or downstream effectors in oncogenic pathways through their interactions with other molecules, such as DNA, RNA and protein [12]. In the cytoplasm, lncRNAs interact with target RNAs, such as mRNAs and microRNAs (miRNAs), or with specific signaling proteins. This affects the expression of target genes, thereby regulating the activities of signaling pathways [12]. In the nucleus, lncRNAs act as the signal, guide, decoy or scaffold to modulate the epigenetic and transcriptional processes, affecting chromatin structure and gene transcription [13,14]. By binding with key components within different signaling pathways, lncRNAs participate in in the regulation of multiple signaling pathways at many levels (epigenetic, transcriptional, post-transcriptional and translational), thus facilitating the formation of complex regulatory networks in tumor cells. 

In this review, we outline the expression, functional cellular roles and underlying molecular mechanisms of lncRNAs in EC progression, highlighting their significant impact on EC-related signaling pathways. Together, we provide an overview of the emerging opportunities and challenges of targeting lncRNA to diagnose and treat EC. 

## 2. Evidence Acquisition

The PubMed database was searched for articles published up to January 2019 using the following keywords: long non-coding RNA, lncRNA, uterine tumor, endometrial carcinoma and endometrial cancer. All studies recognized were assessed for relevance by two authors by checking the title and abstract. All irrelevant articles, studies without access to the full text of the publication, case reports, letters, expert opinions, meeting records, review articles and articles whose methods do not contain biomedical experimental validation were excluded. After this, the full text of any selected article was reviewed by at least two authors. In addition, we also searched the reference lists of selected studies to identify additional relevant articles. 

## 3. Classification of EC Based on LncRNA Expression

An integrative analysis of lncRNAs using TCGA molecular RNA-sequencing profiles of 191 primary endometrioid ECs has identified 858 lncRNAs that were differentially expressed in EC tissues compared to normal endometrium [15]. By applying the clustering of lncRNA expression, these EC patients were classified into three groups: (i) basal-like, (ii) luminal-like and (iii) β-catenin (CTNNB1)-enriched subgroups. The basal-like subgroup was enriched for more aggressive tumors that had a higher pathological grade and TNM stage, with this group showing a higher incidence of *p53* mutation, deletion of PTEN and overexpression of polycomb genes (*EZH2* and *CBX2*). Not surprisingly, this group tends to exhibit poorer survival compared to other groups. The luminal subgroup was associated with the expression of progesterone and estrogen receptor genes, while the β-catenin subgroup was associated with mutations in the *β-catenin* gene and *PTEN* mutation [15]. Overall, 33 lncRNAs (such as HOTAIR) were upregulated, whereas 50 lncRNAs (including LINC00488) were downregulated in the basal-like subgroup as compared to the two other subgroups [15]. These results suggest that analyzing the expression pattern of lncRNAs might be very helpful in distinguishing aggressive endometrioid EC from the non-aggressive disease. 

## 4. Differential Expression and Prognostic Value of LncRNAs in EC

LncRNAs are upregulated or downregulated in ECs compared to normal tissues and their dysregulation has been linked to tumor grade, FIGO stage, the depth of myometrial invasion, lymph node metastasis and patient survival [16,17,18] (Table 1). 

### 4.1. Potential Tumor Suppressive lncRNA

CASC2 expression decreased from normal tissues to EC tissues [19].

### 4.2. Tumor Suppressive lncRNAs

#### 4.2.1. GAS5

GAS5 was downregulated in EC tissues compared with normal tissues and the overexpression of GAS5 in two EC cell lines (HHUA and JEC) resulted in apoptosis in these cells [20]. 

#### 4.2.2. MEG3

The levels of MEG3 were significantly lower in EC tissues than those in adjacent normal tissues [21]. Consistently, two EC cell lines (HEC-1A and KLE) expressed lower levels of MEG3 compared to the normal endometrial cell line ESC [21]. Another study also demonstrated that the expression of MEG3 was significantly lower in EC samples than in normal endometrial tissues [22]. Stable overexpression of MEG3 significantly induced apoptosis and reduced migration and invasion in HEC-1B and Ishikawa cells [22].

#### 4.2.3. FER1L4

FER1L4 was significantly downregulated in EC tissues compared with adjacent normal tissues [23]. Lower levels of FER1L4 was significantly correlated with higher FIGO stages, lymph node metastasis, distant metastasis and worse patient survival [23]. The gain-of-function studies in EC cell line HEC-50 suggested that FER1L4 could inhibit proliferation and induce apoptosis of HEC-50 cells [24]. 

#### 4.2.4. LINC00672

Through qRT-PCR analysis in a total of 176 pairs of ECs and adjacent non-tumor tissues of two distinct Chinese populations, the expression of LINC00672 was shown to be significantly lower in EC tissues than in adjacent normal tissues [25]. A further functional investigation revealed that the overexpression of LINC00672 in HEC-1A and Ishikawa cells reduced cell proliferation [25]. 

### 4.3. Oncogenic lncRNAs

#### 4.3.1. CCAT2

A recent report illustrated that CCAT2 was aberrantly upregulated in EC tissues compared to the normal endometrial tissues [26]. The silencing of CCAT2 suppressed proliferation, migration and invasion of HEC-1-A and RL95-2 cells [26]. 

#### 4.3.2. BANCR

BANCR levels were significantly higher in EC tissues than that in normal endometrial tissues [27]. Increased expression of BANCR was positively correlated with FIGO stage, tumor grade, myometrial invasion and lymph node metastasis in patients with EC [27]. In vitro studies showed that knockdown of BANCR significantly suppressed proliferation, migration and invasion of Ishikawa and HEC-1A cells [27]. 

#### 4.3.3. NEAT1

The levels of NEAT1 were higher in endometrioid EC tissues compared with adjacent normal endometrial tissues [28]. A higher level of expression of NEAT1 was found in those patients with FIGO stage III or IV and with positive lymph node metastasis [28]. Forced overexpression of NEAT1 in HEC-59 cells promoted cell growth and invasion, while knocking down of NEAT1 in HEC-59 cells had the opposite effect [28]. 

#### 4.3.4. MALAT1

Previous evidence suggested that MALAT1 was upregulated in EC tissues compared to their matched adjacent tissues [29]. Ectopic expression of MALAT1 promoted migration and invasion of RL-952 cells [29]. 

#### 4.3.5. H19

Gene expression analysis via in situ hybridization showed that H19 was not expressed in the epithelium of normal endometrium, but is overexpressed in 60% of EC [30]. Similarly, ECs expressed higher levels of H19 than normal endometrial tissues as determined by qRT-PCR analysis [31,32]. H19 expression in EC tissues was noted to progressively increase with tumor grade [32]. Another study showed that the levels of H19 were elevated in mixed endometrioid and serous ECs when compared to pure endometrioid ECs as measured by qRT-PCR analysis, which indicates that H19 may serve as a marker for the more aggressive EC subtype [33]. 

The prognostic value of H19 has been investigated in TCGA EC datasets and those patients with higher H19 expression had a significantly shorter overall survival than those with lower H19 expression [34]. The depletion of H19 in HEC-1B cells and the serous EC cell line ARK2 impaired cell migration and invasion [32,35]. 

#### 4.3.6. HOTAIR

HOTAIR was upregulated in EC tissues as compared to normal endometrial tissues and a higher level of HOTAIR expression was significantly associated with higher tumor grade, positive lymph node metastasis, the depth of myometrial invasion and the presence of lymphovascular space invasion [36]. The patients with a higher level of HOTAIR expression had a significantly poorer overall survival than those with a lower level of HOTAIR expression [36,37]. The depletion of HOTAIR significantly suppressed the proliferation and invasion of HEC-1A cells in vitro and reduced EC tumorigenesis in vivo [38]. HOTAIR was shown to promote viability, migration and invasion by regulating the miR-646/NPM1 pathway in Ishikawa and HEC-1-A cells [39]. 

#### 4.3.7. UCA1

The overexpression of UCA1 was detected in EC tissues and was positively associated with tumor grade, FIGO stage, the depth of myometrial invasion and lymph node metastasis [40]. The silencing of UCA1 attenuated the migration and invasion of HTB-111 and Ishikawa cells [40]. 

#### 4.3.8. Potential Oncogenic lncRNA

Another lncRNA CARLo-5 was significantly upregulated in EC tissues compared to normal tissues and was significantly associated with higher FIGO stages and positive lymph node metastasis [41]. Kaplan-Meier analysis showed that a higher level of CARLo-5 expression was associated with significantly shorter overall survival time in patients with EC [41]. However, the cellular function of CASC2 and CARLo-5 in EC remains to be explored. 

### 4.4. Cancer- or Subtype-Specific lncRNAs in EC

The EC-specific expression pattern observed for H19 supports the notion that H19 may serve as an ideal diagnostic biomarker and interesting potential therapeutic target for EC although further testing will be required. 

While most previous studies have focused on the expression of lncRNAs in type I EC, a recent study reported a lower frequency of focal amplification of the lncRNA OVAL in endometrioid ECs but a higher frequency of focal amplification of OVAL locus in serous ECs [45]. This indicates that OVAL amplification is specific to serous EC. The subtype-specific lncRNA expression patterns need to be further investigated. 

## 5. LncRNAs are Key Regulators of Signaling Pathways in EC

**Figure 1:**
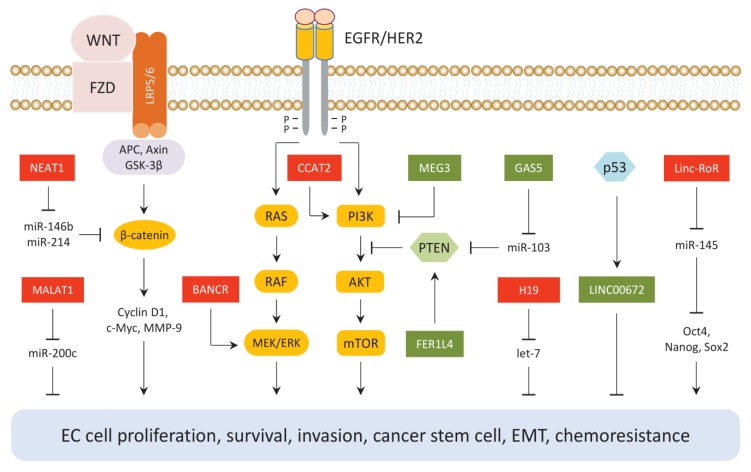
LncRNAs mediate cell signaling in EC cells. Tumor suppressive lncRNAs (GAS5, MEG3, FER1L4 and LINC00672) and oncogenic lncRNAs (CCAT2, BANCR, NEAT1, MALAT1, H19 and Linc-RoR) are upstream modulators or downstream effectors of well-known oncogenic or tumor suppressor pathways in EC, including the PTEN/PI3K/AKT/mTOR, RAS/RAF/MEK/ERK, WNT/β-catenin and p53 signaling pathways. Oncogenic lncRNAs (red) and tumor suppressive lncRNAs (green).

Tumor suppressive lncRNAs (GAS5, MEG3, FER1L4 and LINC00672) and oncogenic lncRNAs (CCAT2, BANCR, NEAT1, MALAT1, H19 and Linc-RoR) have been identified as key regulators of the established tumor suppressor or oncogenic pathways in EC (Figure 1).

### 5.1. The PTEN/PI3K/AKT Signaling Pathway

LncRNA CCAT2 promotes EC cell growth and metastasis by reducing the levels of miR-216b, which is a repressor of the PI3K/AKT pathway [26]. 

Conversely, FER1L4 could enhance PTEN expression and inhibit AKT phosphorylation in EC cells [24]. Another lncRNA GAS5 was also shown to induce PTEN expression by inhibiting miR-103 in EC cells [20]. The results obtained from RNA immunoprecipitation assay showed that MEG3 can combine directly with PI3K protein and reduced its expression, leading to the downregulation of its downstream genes (including mTOR, P70S6K, VEGF-A and BCL-XL) and the attenuation of EC growth in vivo [22]. 

### 5.2. The RAS/RAF/MEK/ERK Signaling Pathway

BANCR promotes EC cell proliferation and invasion via activating the MEK/ERK signaling pathway [27]. 

### 5.3. The WNT/β-Catenin Signaling Pathway

Wnt proteins, which serve as ligands for the Wnt/β-catenin pathway, bind to the Frizzled (FZD) transmembrane receptor and induce the accumulation of nuclear β-catenin. This leads to the downregulation of target genes, such as cyclin D1, c-Myc and MMP-9 [46]. NEAT1 has been suggested to function as an oncogenic sponge in EC where it sequesters several tumor suppressor miRNAs (miR-146b and miR-214) to activate the WNT/β-catenin pathway [47,48]. Moreover, the ectopic expression of MALAT1, a downstream effector of the Wnt/β-catenin pathway [49], promoted EC cell migration and invasion via inhibiting the expression of miR-200c, which suppressed the migration and invasion of EC cells [29]. 

### 5.4. The p53 Signaling Pathway

LINC00672, a direct transcriptional target of p53, was shown to inhibit the development of malignant phenotypes of EC both in vitro and in vivo. This increased the sensitivity of xenograft mice to paclitaxel treatment via serving as a locus-restricted cofactor for p53-mediated gene suppression [25]. 

### 5.5. Epithelial to Mesenchymal Transition and Cancer Stem Cells-Associated Signaling Pathways

In tumors, the epithelial to mesenchymal transition (EMT) was shown to play a critical role in promoting cancer invasion, cancer stemness, immune escape and resistance to therapy [50]. Let-7 is frequently silenced in a variety of tumors, with the ectopic expression of let-7 inhibiting proliferation, EMT and invasion of EC cells and abrogating cancer stem cells (CSCs)-like properties [51,52]. H19 directly binds to let-7 and suppresses its levels in EC cells, resulting in de-repression of let-7 targets (c-Myc, Hmga2 and Imp3) and stimulation of EC cell migration and invasion via inducing EMT [35]. 

Many tumors (including EC) harbor CSCs [53]. CSCs are considered as the primary tumor initiator cells and are resistant to conventional therapies [54]. CSC marker genes, such as Oct4, Sox2, KLF4 and Nanog, are direct targets of miR-145 [55,56]. Interestingly, Linc-RoR, an oncogenic lncRNA, acts as a sponge of miR-145 to induce the expression of Oct4, Sox2 and Nanog in CSC-like EC cells [57], which suggests a mechanism where Linc-RoR could promote CSC properties by negatively regulating the expression of miR-145. 

Numerous studies have reported the link between the dysregulation of lncRNAs and the occurrence of chemoresistance in cancers [58]. HOTAIR induces the resistance of EC cells to cisplatin by inducing autophagy [59]. However, the involvement of lncRNAs in EC chemoresistance is still largely unclear.

## 6. Emerging Role of LncRNA in EC Microenvironment

The tumor microenvironment consists of the extracellular matrix, blood or lymphatic vessels, fibroblasts, immune cells and inflammatory cells [60]. The reciprocal cross-talks between tumor cells and tumor microenvironment can protect tumor cells from immune evasion or nutrient deficiency, both of which are critical for driving tumor progression and resistance to treatment [60]. 

**Figure 2:**
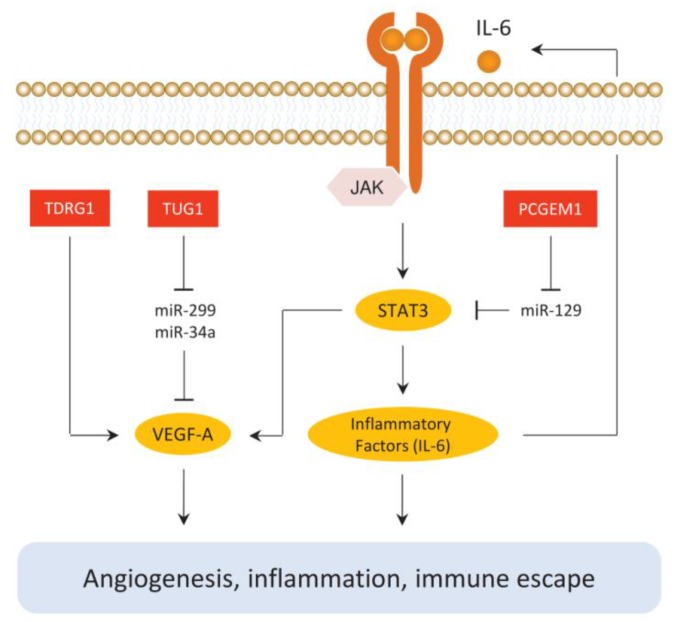
LncRNAs are mediators of angiogenesis and inflammation in EC. TUG1 and TDRG1 stimulate the VEGF-A pathway. PCGEM1 is implicated in activating the JAK/STAT3 pathway.

The regulatory roles of lncRNA during EC progression are not limited to tumor cells as they are also implicated in generating a tumor-promoting microenvironment (Figure 2). 

Angiogenesis, the process of the formation of new blood vessels, is controlled by many angiogenic factors, such as the VEGF family [61]. VEGF-A is one of the most potent and specific endothelial cell growth factors and plays important roles in the growth of EC [6]. In EC cells, TDRG1 was significantly upregulated in ECs compared to normal endometrial tissues. This could directly bind to VEGF-A protein and upregulated its expression, thus promoting EC proliferation, invasion and migratory ability in addition to inhibiting cell apoptosis [42]. In addition, TUG1 expression in EC tissues was significantly higher than that in adjacent normal tissues, which can enhance the progression of EC via enhancing the expression of VEGF-A, possibly through inhibiting miR-34a and miR-299 expression [43]. 

Tumor-associated inflammation is both a consequence and a driver of tumorigenesis [62]. NF-κB and STAT3 are two major factors controlling tumor inflammation, angiogenesis and invasiveness [63]. The STAT3 pathway can be induced by cytokines, such as IL-6 [64]. The activation of the JAK/STAT3 pathway results in the nuclear translocation of STAT3 and subsequent translation of key downstream target genes, including cyclin D1, Bcl-xL, c-Myc, Mcl-1 and VEGF-A [65]. Importantly, STAT3 induces the expression of many cytokines, chemokines and other mediators, such as interleukin-6 and cyclooxygenase 2. In turn, this activates STAT3, thus forming autocrine and paracrine feed-forward loops that promote tumor inflammation [66]. The levels of PCGEM1 were increased in EC tissues, which was shown to promote the proliferation, migration and invasive ability of EC cells through a STAT3-dependent mechanism. In this mechanism, PCGEM1 indirectly increases the levels of STAT3 through inhibiting miR-129 expression while the elevated STAT3 expression further upregulates the expression of Survivin, VEGF-A and MMP-2 [44]. 

PD-L1 is a powerful immunosuppressive molecule that inactivates the function of tumor-specific T cells by binding to its receptor PD-1 on T cells [67]. PD-L1 expression was detected in 48% of ECs and a higher level of PD-L1 expression was significantly associated with lymph node metastasis [68]. A recent analysis of the lncRNA-miRNA-mRNA network based on competitive endogenous RNA reveals that H19 has the potential to induce the expression of PD-L1 in laryngeal squamous cell carcinoma, which possibly occurs by modulating a set of miRNAs (including miR-214) [69]. The involvement of lncRNAs in regulating PD-L1 or other immune inhibitory molecules in EC cells should be explored and the identification of such mechanisms would deepen our understanding of the molecular basis underlying the immune escape mechanisms of EC and facilitate more choices when designing immunotherapy against EC. 

## 7. Future Perspectives: Hope and Challenges

To date, most investigations have centered on the in vitro EC cell line models with less aggressive potential and as a result, very little is known about the role of lncRNA in high-grade endometrioid or serous EC. The lncRNA expression pattern that differentiates the aggressive EC from non-aggressive EC is still unknown. 

In addition to aberrant expression, single-nucleotide polymorphisms (SNPs) within the lncRNA transcripts or their promoters or mutations within the non-coding genome may dramatically alter the secondary structure, expression and function of these lncRNAs, thereby exerting important effects in cancer [70]. Genotype–phenotype correlation studies showed that the G allele of rs6983267, a reported oncogenic SNP at 8q24.21 [71], was significantly correlated with a higher level of CARLo-5 expression and poorer overall survival in EC patients [41]. The rapid advance in state-of-the-art next-generation sequencing techniques, re-analysis of the publicly available databases and the progress in bioinformatics computing methods would facilitate large-scale discovery of SNPs and mutations in lncRNAs and help us to identify particularly important EC-associated lncRNAs for future study [72,73,74,75]. 

Extracellular vesicles (EV; including exosomes and microvesicles) can carry proteins, lipids, miRNA, mRNA, DNA and recently lncRNA from one cell to another [76]. Exosomes released by tumor cells have the potential to induce malignant phenotypes of recipient cells [77,78]. As an example, EVs secreted from invasive breast cancer cell subpopulations were demonstrated to upregulate the proliferative, migratory and angiogenic potential of the parent population [77]. Moreover, EVs released from drug-resistant cancer cells was shown to transmit the anti-cancer drug resistance to other cancer cells [79]. A recent study demonstrated that EC cells could transmit miRNAs to endometrial fibroblasts via exosomes [80]. These results suggest that the transfer of ncRNAs (such as miRNA or lncRNA) via EVs may represent a new mechanism for intercellular communication that promotes the growth and metastasis of EC. 

Once released, exosomes enter the circulation and can be isolated from blood, urine, saliva and ascites [81], indicating that tumor cell-derived EVs in circulating bodily fluids are readily accessible and can act as non-invasive specimens for biomarker discovery. The level of EVs bearing a candidate protein was elevated before diagnosis by conventional imaging techniques and the presence of EVs bearing candidate mRNAs was a predictor of progression-free survival in several cancers [82,83]. These findings have suggested that EVs released by tumor cells may be used for early disease detection and disease monitoring. A potential direction for future research is to explore the use of lncRNAs within EC cell-derived EVs for disease diagnosis and monitoring [84]. 

However, there are some challenges to be solved before the tumor cell-derived EVs can be used as biomarkers in a clinical setting. The accurate quantification of circulating lncRNA in exosomes faces several challenges, such as the correct procedure for specimen collection and handling, and the proper selection of internal controls for data normalization [85,86]. 

Given the importance of lncRNAs in tumors, multiple approaches, such as knockdown pathogenic lncRNA using siRNA, prevention of the binding of a lncRNA with its binding partners or creating lncRNA-binding small molecules have been developed [3,87,88]. Hopefully, the in vitro and in vivo studies have shown that RNA interference targeting oncogenic lncRNAs (including BANCR, H19, HOTAIR, UCA1, SNHG8, ASLNC04080 and PVT1) [27,35,38,40,89,90,91] or enforced expression of tumor suppressor lncRNA MEG3 could exert promising anti-tumor effects on EC [21]. 

Recently, the utilization of genome-editing with CRISPR/Cas9 system represents a promising opportunity for designing effective anti-cancer therapy [92]. Targeting UCA1 via specifically designed gRNAs of CRISPR/Cas9 system effectively inhibited the proliferation, migration and invasion of bladder cancer cells in vitro and suppressed the growth of xenograft tumors from bladder cancer cells in nude mice [93]. 

However, in a genome-wide analysis, only 38% of 15929 lncRNA loci are safely amenable for CRISPR applications, while the remaining lncRNA loci are at risk to inadvertently deregulate neighboring genes [94], suggesting that targeting some but not all lncRNA regions via the CRISPR/Cas9 system is challenging [95]. Several techniques, such as SpCas9-HF1, a high-fidelity variant of SpCas9 that contains alterations in the amino acid sequence, have been utilized to reduce the off-target effects due to CRISPR/Cas9 [96,97]. Moreover, a tumor-specific promoter may be used to drive Cas9 expression, thus providing a relatively high specificity of CRISPR/Cas9 for cancer cells [98]. 

## 8. Conclusions

By employing three basic modules, which are namely RNA–RNA, RNA–protein and RNA–DNA interactions, lncRNAs form both transcriptional and post-transcriptional regulatory networks, which play the pivotal roles in modulating the malignant phenotypes of tumor cells and the remodeling of the tumor microenvironment. Tumor suppressive lncRNAs (GAS5, MEG3, FER1L4 and LINC00672) and oncogenic lncRNAs (CCAT2, BANCR, NEAT1, MALAT1, H19, Linc-RoR, TUG1, TDRG1 and PCGEM1) may be a few such examples. Since lncRNAs can be detected in both cancer samples and various bodily fluids and strongly resist RNases, they have considerable diagnostic value and might be hopeful biomarkers for early detection and disease monitoring of EC. H19 exhibits highly EC-specific expression and might be a potential new target for EC treatment. Further clarification of lncRNA-mediated regulatory networks will shed light into the mechanisms behind EC and new studies continue to suggest novel lncRNAs as possible diagnostic markers and therapeutic targets. 

## Figures and Tables

**Table 1:** The expression, function and mechanism of lncRNAs in EC.

LncRNA	Expression	Function	Mechanism	Ref.
CASC2	Downregulation	—	—	[19]
GAS5	Downregulation	Tumor suppressor	Induced PTEN expression via inhibiting miR-103	[20]
MEG3	Downregulation	Tumor suppressor	Combined directly with PI3K protein and reduced its expression	[21,22]
FER1L4	Downregulation	Tumor suppressor	Enhanced PTEN expression	[23,24]
LINC00672	Downregulation	Tumor suppressor	Served as a locus-restricted cofactor for p53-mediated gene suppression	[25]
CCAT2	Upregulation	Oncogene	Activated the PI3K/AKT pathway by reducing miR-216b expression	[26]
BANCR	Upregulation	Oncogene	Activated the MEK/ERK signaling	[27]
NEAT1	Upregulation	Oncogene	Activated the WNT/β-catenin pathway via inhibiting miR-146b and miR-214 expression	[28]
MALAT1	Upregulation	Oncogene	Inhibited miR-200c expression	[29]
H19	Upregulation	Oncogene	Induced EMT via inhibiting let-7 expression	[30,31,32,33,34,35]
HOTAIR	Upregulation	Oncogene	Increased NPM1 expression via suppressing miR-646 expression	[36,37,38,39]
UCA1	Upregulation	Oncogene	—	[40]
CARLo-5	Upregulation	—	—	[41]
TDRG1	Upregulation	Oncogene	Directly interacted with VEGF-A protein and upregulated its expression	[42]
TUG1	Upregulation	Oncogene	Increased VEGF-A expression via suppressing miR-34a and miR-299	[43]
PCGEM1	Upregulation	Oncogene	Activated the STAT3 pathway via reducing miR-129 expression	[44]
